# Dual Functions of Interferon Regulatory Factors 7C in Epstein-Barr Virus–Mediated Transformation of Human B Lymphocytes

**DOI:** 10.1371/journal.pone.0009459

**Published:** 2010-03-04

**Authors:** Yong Zhao, Dongsheng Xu, Yanjun Jiang, Luwen Zhang

**Affiliations:** 1 School of Biological Sciences, University of Nebraska, Lincoln, Nebraska, United States of America; 2 Nebraska Center for Virology, University of Nebraska, Lincoln, Nebraska, United States of America; Karolinska Institutet, Sweden

## Abstract

Epstein-Barr virus (EBV) infection is associated with several human malignancies. Interferon (IFN) regulatory factor 7 (IRF-7) has several splicing variants, and at least the major splicing variant (IRF-7A) has oncogenic potential and is associated with EBV transformation processes. IRF-7C is an alternative splicing variant with only the DNA-binding domain of IRF-7. Whether IRF-7C is present under physiological conditions and its functions in viral transformation are unknown. In this report, we prove the existence of IRF-7C protein and RNA in certain cells under physiological conditions, and find that high levels of IRF-7C are associated with EBV transformation of human primary B cells in vitro as well as EBV type III latency. EBV latent membrane protein 1 (LMP-1) stimulates IRF-7C expression in B lymphocytes. IRF-7C has oncogenic potential in rodent cells and partially restores the growth properties of EBV-transformed cells under a growth-inhibition condition. A tumor array experiment has identified six primary tumor specimens with high levels of IRF-7C protein—all of them are lymphomas. Furthermore, we show that the expression of IRF-7C is apparently closely associated with other IRF-7 splicing variants. IRF-7C inhibits the function of IRF-7 in transcriptional regulation of IFN genes. These data suggest that EBV may use splicing variants of IRF-7 for its transformation process in two strategies: to use oncogenic properties of various IRF-7 splicing variants, but use one of its splicing variants (IRF-7C) to block the IFN-induction function of IRF-7 that is detrimental for viral transformation. The work provides a novel relation of host/virus interactions, and has expanded our knowledge about IRFs in EBV transformation.

## Introduction

Epstein-Barr virus (EBV) infection has been associated with the development of several human malignancies, including nasopharyngeal carcinoma (NPC), Burkitt's lymphoma (BL), Hodgkin's lymphoma, T cell lymphoma, and gastric carcinoma [1], [2]. In immunocompromised individuals, such as organ transplant recipients and AIDS patients, EBV almost certainly triggers two fatal cancers without the necessity for cofactors: AIDS-associated central nervous system (CNS) lymphoma and post-transplantation lymphoproliferative disorder (PTLD) [3].

EBV establishes several distinctive types of latencies in host cells. In type I latency, EBV nuclear antigen 1 (EBNA1) and small EBV-encoded, non-polyadenylated nuclear RNAs (EBER-1 and -2) are expressed in host cells. In contrast, six nuclear proteins (EBNA-1, EBNA-2, EBNA-3A, EBNA-3B, EBNA-3C, and EBNA-LP), three membrane proteins (latent membrane protein 1 [LMP-1], LMP-2A, and LMP-2B), plus EBERs are expressed in type III latency [1], [2].

EBV transforms adult primary B cells into continually growing lymphoblastoid cell lines (LCLs) and concomitantly establishes type III latency *in vitro*. LMP-1 is required for the transformation process of B lymphocytes [4], [5], [6]. LMP-1 acts as a constitutively active, receptor-like molecule that does not need the binding of a ligand [7], and appears to be a central effector of altered cell growth, survival, adhesive, invasive and antiviral potential [8], [9], [10], [11], [12], [13], [14].

Interferon (IFN) regulatory factors (IRF) are a small family of transcription factors with multiple functions [15], [16], [17], [18], [19], [20], [21], [22]. IRF-7 was first cloned in the context of EBV latency through its binding to an EBV latency promoter (Qp), which regulates expression of EBNA1 [23]. IRF-7 is predominantly expressed in the spleen, thymus and primary blood lymphocytes (PBL) as a lymphoid-specific factor [23]. Currently it is established that IRF-7 has four splicing variants designated as IRF-7A, IRF-7B, IRF-7C, and IRF-7H [23], [24]. By now, most of published papers about IRF-7 focused on IRF-7A, the major splicing variant. Other than in the negative regulation of viral promoter (Qp), IRF-7 also positively regulates cellular Tap-2 as well as viral LMP-1 promoters in EBV latency [25], [26]. IRF-7 and EBV has intimate relations: EBV both induces the expression of and activates (by phosphorylation and nuclear translocation) IRF-7 in viral latency program [21], [27]. In addition, IRF-7 is associated with EBV-transformed CNS lymphomas and has oncogenic properties [28]. Other than functioning in EBV transformation, IRF-7 is a master gene involved in the activation of type I IFN genes that are the key mediators of the host innate immunity [29].

In this report, we have studied the functions of a splicing variant of IRF-7. IRF-7C is an alternative splicing variant that the original open-reading frame of IRF-7 has been changed. IRF-7C has only the DNA binding domain with 165 amino acids and unique 13-amino acids at the C-terminus [23]. Whether IRF-7C is present under physiological conditions is unknown. Here, we have generated an IRF-7C-specific antibody and find that IRF-7C is associated with EBV transformation of primary B lymphocytes *in vitro* and is highly expressed in some human primary lymphomas *in vivo*. IRF-7C is expressed proportionally to other IRF-7 splicing variants, and IRF-7C blocks the activation of IFN by IRF-7. Because IRF-7 is activated in EBV transformation and activated IRF-7 is known to induce the expression of IFNs, which is detrimental to transformation. Thus, EBV may use both IRF-7 splicing variants for its oncogenic transformation, but uses one of its splicing variants (IRF-7C) to block the detrimental IFN-induction function of IRF-7.

## Materials and Methods

### Plasmids and Antibodies

Expression plasmids of IRF-7C, LMP-1, or its signaling defective mutant, LMP-DM, and full-length IRF-7 antibody were described previously [23], [30]. IRF-7CD was made by a PCR to remove the last 13-amino acids of IRF-7C, and cloned into pcDNA3 vector. IRF7C-K92E plasmid was obtained with the use of a PCR mutagenesis kit (Invitrogen). LMP-1 Ab (CS1-4) antibodies were purchased from DAKO. GAPDH (0411) antibody was purchased from Santa Cruz Biotechnology. Tubulin antibody was purchased from Sigma. Rabbit IRF-7C antibody was obtained by synthesizing the polypeptide with the last C-terminus15 amino acid sequence of the IRF-7C, including the unique 13 amino acid sequence that is not present in other three splicing variants IRF-7A, -7B and -7H. The peptide was conjugated to a carrier and used as the target for antibody production (Pacific Immunology Corp).

### Cell Culture, Interferons, and Sendai Virus

293T is a human fibroblast line (from ATCC) and were grown in Dulbecco's modified Eagle medium (DMEM, Gibco BRL) supplemented with 10% fetal bovine serum (FBS; Gibco BRL) and 1% Penicillin-streptomycin (PS) at 37°C in a 5% CO_2_ incubator. DG75 is an EBV-negative Burkitt's lymphoma (BL) cell line [31]. BL41 is an EBV-negative BL line, BL41-EBV was generated by *in vitro* infection of BL41 with EBV B95-8 strain [32]. Sav I, Sav III are genetically identical cell lines that differ only in their latency types [33]. The THP-1 cell line (Human acute monocytic leukemia cell line) is derived from the peripheral blood of a 1 year old human male with acute monocytic leukemia [34]. These cells were maintained in RPMI-1640 plus 10% fetal bovine serum (FBS). Primary B cells and EBV-transformed B cell lines are described [35]. Primary B cells isolation was done as described with the use of CD19 antibody conjugated magnetic beads (Dynal Inc) [28]. NIH3T3 cells were purchased from ATCC, and maintained in DMEM plus 10% Calf Bovine Serum (CBS). Recombinant human IFN-α was purchased from R & D Systems. 100 units of IFN were used for the treatment of cells. Sendai virus stock was purchased from Spafas, Inc. For virus infection, 200 HA units/ml Sendai virus were added to the target, and cells were then collected for RNA isolation.

### Transfection, Growth and Reporter Assays

Transfection of IB4 cells were achieved by using Amaxa Nucleofector Device. The 1×10^6^ cells were transfected with 5 µg of DNA in solution B and program U20. Transfected cells were immediately put into 12 wells plates with RPMI plus FBS. Approximately 70% of cells could be transfected with the protocol. One day later, live cells were isolated by Ficoll-Paque Plus (GE Healthcare) following manufacturer's recommendations. The live cells were counted and dispensed in culture flask at 3.5×10^5^ cells/ml: this was counted as Day 1 after transfection. Everyday, a small portion of cells were stained with trypan blue and live cells were counted using the hemocytometer. Statistical analyses of the differences were determined by paired t test with GraphPad Prism software version 5.0 (GraphPad Software, Inc., San Diego, CA).

Electroporation (320 V; 925 microfarads) was used for transfection of the DG75 cells as described previously [13], [14], [36]. A total 5 µg of DNA was used for transfection of DG75 cells. 1 µg of LMP-1-expression plasmids were always used in transfection because similar LMP-1 expression levels in transfected and EBV type III latency cells could be achieved under such conditions. Enrichment for CD-4-positive cells was performed with the use of anti-CD-4-antibody conjugated to magnetic beads according to the manufacturer's recommendation (Dynal, Inc.). DG75 cells were transfected with CD-4 expression and other plasmids. One day after the transfection, the cells were used for isolation of CD-4-positive cells with the use of Dynabeads CD4 (Dynal Inc.) The transfected cells were incubated with Dynabeads-CD4 at 72 µl of beads/10^7^ cells for 20–30 min at 4°C with gentle rotation. CD4-positive cells were isolated by placing the test tubes in a magnetic separation device (Dynal magnet). The supernatant was discarded while the CD4-positive cells were attached to the wall of the test tube. The isolated cells were used to extract total RNAs or prepare cell lysates immediately.

293T cells were seeded and grown to 40 to 50% confluence in each well of 12-well dishes (Becton Dickinson labware). For each well, 293T cells were transfected with 0.2 ug of total DNA mixed with 4 ul Effectene according to the manufacture's instructions. Twenty four hours after transfection, the cells were collected by centrifuging at 800×g for 5 mins, and washed once with phosphate-buffered saline (1x PBS). Luciferase activities were measured using a luciferase assay system (Promega) according to the manufacturer's procedure. Data were averaged from the results of multiple transfections performed in at least three independent experiments.

### Western Blot Analysis with Enhanced Chemiluminescence (ECL)

Separation of proteins on SDS-PAGE and western blot were carried out following standard protocol as described [28], [37].

### RNA Extraction and RNase Protection Assays (RPA)

Total RNA was isolated from cells using the RNeasy total RNA isolation kit (Qiagen, Valencia, CA) or TRIzol extraction. RPA was performed with 10 µg of total RNA using the RNase protection assay kit II (Ambion, Houston, TX) at 55°C. Sometimes, gradient temperatures were performed for RPA when difficulties in RPA were encountered [38]. The GAPDH probe was from U. S. Biochemicals. The probe for IRF-7 (for all splicing variants) was a described before. To prepare the IRF-7C-specific RPA probe, the specific region of IRF-7C was amplified by PCR using oligonucleotide primers (5′-GGGGTACCCTACTGCCCACCCGTACAGC-3′ and 5′-CGGGAATTCGAGGCTGAGACTGCGGAGCG-3′) and cloned into pcDNA3 vector (Invitrogen). The plasmid was digested by EcoRI, and transcribed with T7 RNA polymerase. This template would produce a 251-nucleotide run-off transcript encompassing the splicing junction region.

### NIH 3T3 Cell Transformation Assays

Subconfluent cultures of NIH 3T3 cells seeded in 60-mm-diameter tissue culture dishes were transfected with 500 ng of desired expression plasmids by the calcium phosphate method. Two days after transfection, the cultures were subcultured into growth medium supplemented with G418 to select for drug-resistant stably transfected cell populations. NIH 3T3 cells stably expressing target proteins were pooled and single-cell suspensions (5×10^4^ cells per 60-mm-diameter dish) of each cell line were suspended in growth medium supplemented with 0.3% agar. The appearance of proliferating colonies of cells was monitored and quantitated for up to 4 weeks.

### Human Tumor Array Analysis

Slides containing multiple tumor specimens were purchased from NIH Tissue Array Research Program (TARP). Routine immunostaining protocols were employed for array slides (TARP3) [28]. The TARP3 slides contain 441 primary tumor specimens and 50 normal tissues. The primary antibody used for this study is our polyclonal IRF7C-specific antibody generated in our laboratory. A Cy-2-labeled donkey anti-rabbit secondary antibody was purchased from The Jackson Laboratory. Propidium iodide was from Sigma and used to stain the nuclei. The slides were examined by confocal microscopy in UNL Microscope Facility.

## Results

### IRF-7C Is Associated with EBV Type III Latency

**Figure 1:**
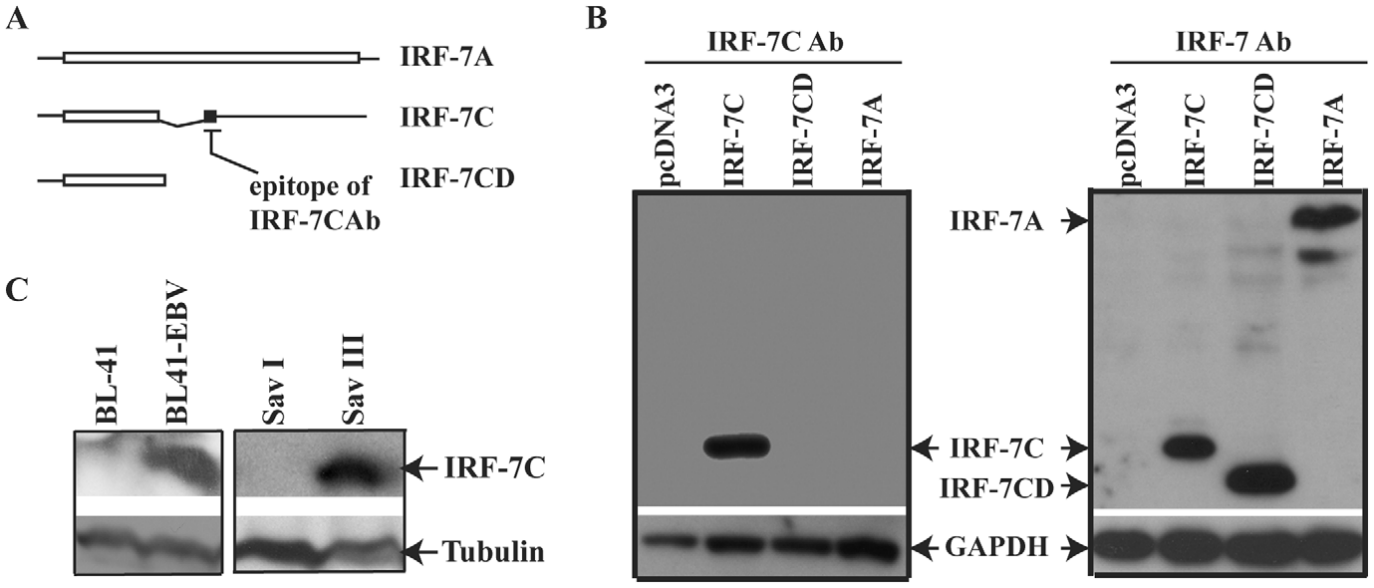
IRF-7C protein is associated with type III latency. A. Schematic diagram of IRF-7C-specific epitope. The open bar represents open-reading frames (ORF). The expression plasmids were made as shown. The IRF-7C has unique 13 amino-acid (aa), represented by solid bar, at the C-terminus because the splicing changes the original ORF. The peptide was synthesized according to the last 15-aa sequence in IRF-7C and was used for antibody production. IRF-7CD is an expression plasmid that lacks the C-terminal 13-aa of IRF-7C. Specific epitope for IRF-7C is shown. The drawing is not on scale. B. Examination of IRF-7C-specific antibody. 293T cells were transfected with various expression plasmids as shown on the top. The expression levels of IRF-7C and GAPDH proteins were determined by Western blotting. Left panel: IRF-7C-specific antibody was used. Right panel: full-length IRF-7 antibody was used. C. IRF-7C protein is highly expressed in cells with EBV type III latency. Cell lysates from the indicated cells lines were separated by 12% SDS-PAGE. The expression levels of IRF-7C and tubulin proteins were determined by Western blotting. The images in the same box indicate that they are derived from the same membrane. The identities of proteins are shown.

A short ORF and long 3-UTR sequence are characteristic features to trigger a nonsense-mediated mRNA decay (NMD) degradation pathway [39]. IRF-7C RNA structure fits the characters. It is thus important to test if IRF-7C is indeed expressed in cells. The current available IRF-7 antibodies cannot distinguish all IRF-7 splicing variants. In order to study the function of IRF-7C, we have made an antibody specifically for IRF-7C C-terminal 13-amino acid peptide (Figure 1A, see Materials and Methods for details). As shown in Figure 1B, while full-length IRF-7 antibody detected all IRF-7 splicing variants, the IRF-7C peptide antibody only detected IRF-7C protein. Thus, the antibody is indeed specific to IRF-7C.

Next, we examined expression of IRF-7C in EBV-positive cell lines. BL41, BL41-EBV, Sav I and Sav III were chosen as cell lines for this analysis. These are genetically identical lines: BL41 is an EBV-negative BL line and BL41-EBV is its EBV-infected counterpart with type III latency. Sav I and SavIII have identical genetic background. Save I is a type I latency while Sav III is a type III latency cell line. As shown in Figure 1C, IRF-7C protein is highly expressed in EBV type III latency cells (BL41-EBV and Sav III).

**Figure 2:**
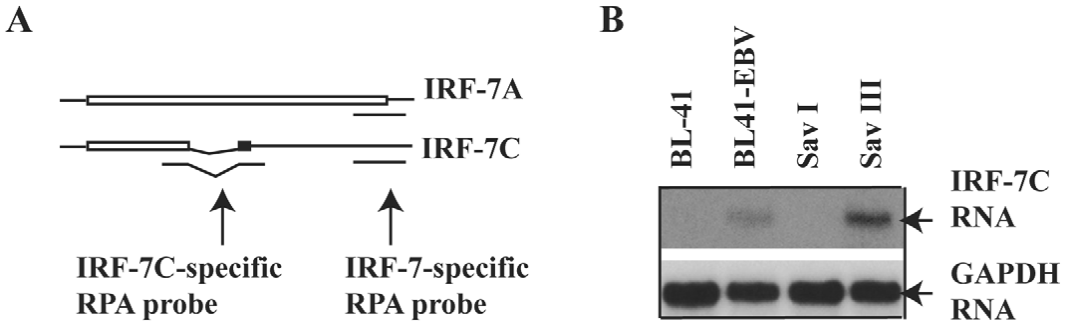
Expression of IRF-7C RNA is associated with type III latency. (A) Schematic diagram of IRF-7C-specific RPA probe. The bar represents ORF. The IRF-7C-specific RPA probe encompasses the splicing junction. The IRF-7-specific probe region for all splicing variants is also shown. The drawing is not on scale. (B) IRF-7C RNA is highly expressed in cells with EBV type III latency. Total RNAs from the indicated cells lines were used for RPA with IRF-7C and GAPDH-specific probes. The images in the same box indicate that they are derived from the same gels. The identities of RNAs are shown.

To confirm the association data in Figure 1, we have tested the RNA expression levels of IRF-7C in these cell lines. A specific RPA probe was designed for specific detection of the expression of IRF-7C RNA (Figure 2A, see Materials and Methods for details). RPA was performed with the IRF-7C-specific plus GAPDH probes. As shown in Figure 2B, IRF-7C RNA was expressed at much higher level in BL41 EBV and Sav III, which is in consistent with protein expression data (Figure 1C). The expression of all IRF-7 splicing variants is also similar as reported previously (data not shown, and references [26], [30]). Thus, the expression of IRF-7C is associated with EBV type III latency at both RNA and protein levels.

### IRF-7C Is Associated with EBV Transformation Processes

**Figure 3:**
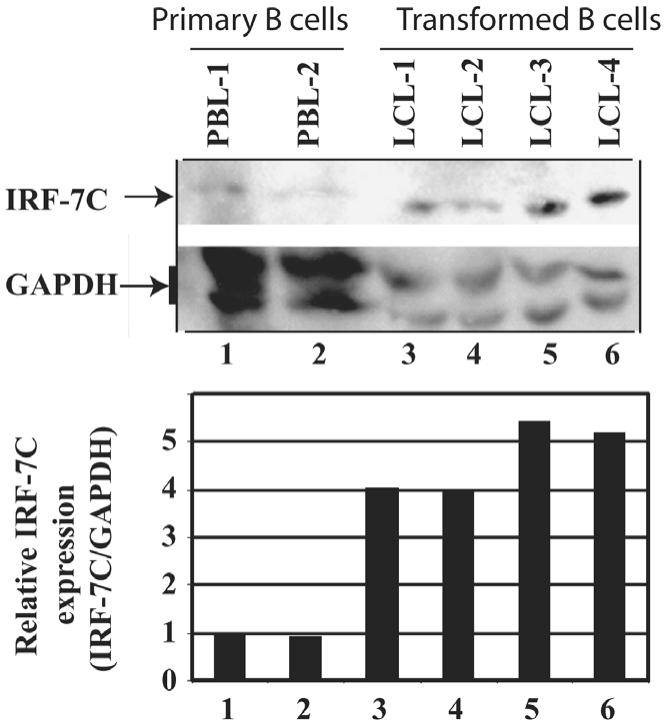
IRF-7C is associated with EBV transformation of primary B lymphocytes in vitro. Top panel: IRF-7C is highly expressed in EBV-transformed cells. Primary B cells were isolated from fresh human blood by the use of CD19 antibody-conjugated magnetic beads. Lysates from primary B cells from two individuals (lanes 1 and 2) and EBV-transformed B cell lines (lanes 3–6) were separated by 12% SDS-PAGE. The expression levels of IRF-7C and GAPDH were determined by Western blotting. The same membrane was stripped and reprobed with GAPDH antibody. Bottom panel: relative levels of IRF-7C expression in EBV transformed cells. The relative levels of IRF-7C expression (IRF-7C/GAPDH) were obtained by measuring intensity of IRF-7C and GAPDH from Panel A using ImageJ 1.37v software (NIH).

Because IRF-7 has been shown to be a potential factor in EBV transformation processes, we thus examined if IRF-7C is involved in EBV transformation processes. Primary B cells were isolated from fresh blood by CD19-antibody-conjugated magnetic beads. The expression of IRF-7C from primary B cells of different individuals was compared with EBV-transformed B cell lines in vitro (LCL). As shown in Figure 3, high levels of IRF-7C expression are associated with EBV-transformed cells. The same cell lysates were also used for examination of other proteins: the expression of IRF-1, -2, and -3, STAT-2, and -3 is apparently not associated with this process [28], [35]. The expression of LMP-1 in the EBV transformed cells had been examined in our previous publication [28]. Thus, IRF-7C protein is stimulated during EBV transformation in vitro. Because EBV-transformed LCLs are in type III latency, these data also reinforce the previous notion that IRF-7C is associated with type III latency.

### LMP-1 Stimulates the Expression of IRF-7C

**Figure 4:**
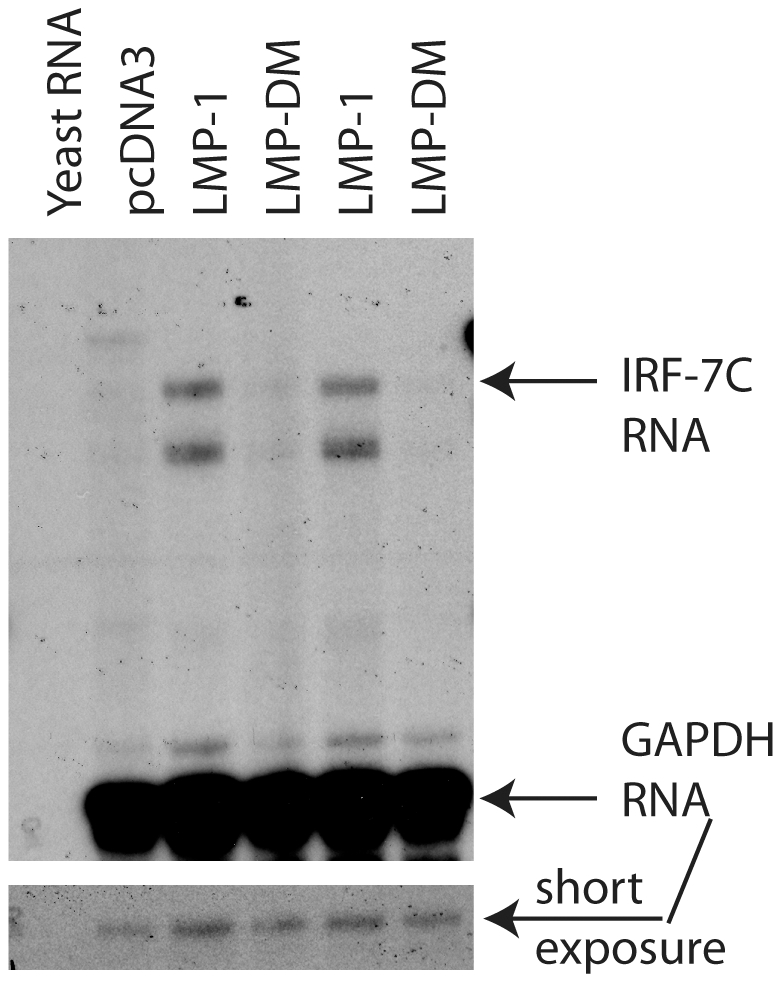
LMP-1 stimulates the expression of IRF-7C. DG75 cells were transfected with pcDNA3, LMP-1, or LMP-DM expression plasmids. The transfected cells were isolated, and total RNAs were isolated and used for RPA with IRF-7C and GAPDH-specific probes. Yeast RNA was used as negative control. Specific protections of IRF-7C and GAPDH RNAs are indicated.

Because LMP-1 is the primary inducer of IRF-7, we tested if LMP-1 is responsible for the induction of IRF-7C. EBV-negative DG75 cells were transfected with LMP-1 or LMP-DM and a CD4-expression plasmid. LMP-DM is a mutant of LMP-1 that fails to activate major intracellular signals [30]. The levels of IRF-7C were determined by RPA after selection of the transfected cells by the use of CD-4 antibody-conjugated magnetic beads (see Materials and Methods for details). As shown in Figure 4, LMP-1 expression causes a marked increase in IRF-7C RNA levels in DG75 cells; however, LMP-DM was not. Therefore, LMP-1 is probably responsible for the induction of IRF-7C in EBV type III latency cells.

### IRF-7C Has Oncogenic Potential

**Figure 5:**
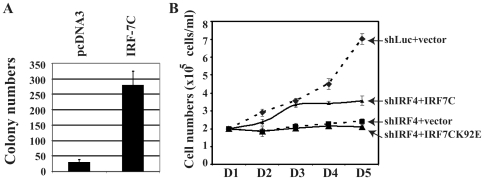
IRF-7C has oncogenic potential. (A) IRF-7C causes anchorage-independent growth of NIH 3T3 cells. A soft-agar assay was used for an IRF-7C stably expressing cell line established in NIH 3T3 cells. The numbers are averages from three independent experiments and standard deviations are also shown. (B) IRF-7C partially restores the growth property of IRF-4-knockdown cells. Knockdown of IRF-4 inhibits the growth of EBV-transformed cells. An EBV-transformed cell line (IB4) was transfected with shLuc, shIRF4, shIRF4 plus IRF-7C, or shIRF4 plus IRF7C-K92E by use of an Amaxa Nucleofector device respectively. One day after transfection, live cells were isolated and seeded. At the indicated days after transfection, surviving cells were enumerated by trypan blue exclusion. Each point represents the number of live cells (mean ± standard deviation) from three different counts. The difference between shIRF4, and shIRF4+IRF-7C is statistically significant (p = 0.0071). One representative of three independent experiments is shown.

Because of the fact that IRF-7 has oncogenic potential, we would like to test if IRF-7C also has a similar property. NIH3T3 cells were chosen for the analyses. IRF-7C expression plasmid was transfected into NIH 3T3 cells, and the IRF-7C-expressing cells were selected in G418-containing medium. Stable cell lines were pooled, and the expression of IRF-7C was confirmed (data not shown). While vector control lines showed a limited numbers of colonies, IRF-7C could induce the growth of NIH 3T3 cells in soft agar assay (Figure 5A). These data indicate that IRF-7C has oncogenic potential based on this assay system. We also did the comparative studies on the potency of transformation with various IRF-7 splicing variants. However, the expression of IRF-7C was always higher that that of IRF-7A (data not shown). Thus, we were unable to conclude on which splicing variant is more potent in oncogenesis.

### IRF-7C Partially Rescues the IRF-4-Knockdown-Mediated Growth Inhibition

Because we have not established a system to knockdown endogenous IRF-7 specifically, we could not test the role of IRF-7C in EBV transformation directly without other IRF-7 splicing variants. However, we have previously shown that IRF-4-knockdown caused growth inhibition in EBV-transformed cells [40]. Because all IRFs bind to similar DNA sequences, and IRF-7C has DNA binding domain, we tested if IRF-7C could rescue the IRF-4-knockdown-mediated cell growth inhibition. The expression of IRF-4 in IB4 cells could be specifically inhibited with transfection of three different siIRF4 expression plasmids together, and the growth of these cells was significantly inhibited. Once IRF-7C was co-transfected, the siIRF4-mediated growth inhibition was partially relieved (Figure 5B). And an IRF-7C mutant, IRF-7CK92E had no effects. The IRF7C-K92E was unable to bind to DNA based on EMSA assay ([36], and Data not shown). These data suggest that IRF-7C has an effect on IRF-4-knockdown-mediated growth inhibition.

### IRF-7C Is Expressed in Lymphoma Specimens

**Figure 6:**
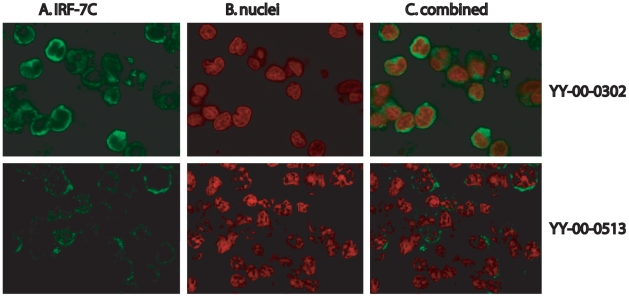
Expression of IRF-7C in human tumor specimens. The IRF-7C expression in tumor arrays was detected by staining with the IRF-7C-specific antibody and a Cy-2-labeled donkey anti-rabbit secondary antibody, followed by examination in confocal microscope. Propidium iodide was used to stain the nuclei. The colors were artificially mounted to facilitate viewing. Panel A: IRF-7C in green; Panel B: nuclei in red; and Panel C shows the combination of IRF-7C and nuclei. Two tumor specimens are shown: top: YY-00-0302; bottom: YY-00-0513.

Because we have the specific antibody against IRF-7C, the expression of the IRF-7C protein in various tumors was examined in a human tumor array from NCI Tissue Array Research Program (TARP). The tumor slides (TARP3) contained normal and various tumor specimens (total: 491 specimens). IRF-7C was predominantly expressed in several tumor specimens (Figure 6), but not in other tumor specimens or normal tissues. In these tumors, IRF-7C-positive cells are over 25% in tumor areas. Surprising, these IRF-7C-positive specimens are all lymphomas with the limited information provided by TARP3 (Table 1). Some other specimens also showed sporadic IRF-7C-positive cells (data not shown). Thus, IRF-7C is apparently highly expressed in some primary tumors, although the linkage of IRF-7C and EBV in vivo has not yet been established in this study.

**Table 1:** Expression of IRF-7C in human tumor specimens.

Block Code	Tumor Type	Sex	Age	Other Information
YY-00-0513	Lymphoma	M	59	IPOX positive CD5, CD20, CD23
YY-00-0509	Lymphoma	M	54	No information
YY-00-0126	Lymphoma	F	74	Flow, positive for CD10, CD19, CD20, lambda
YY-00-0135	Lymphoma	F	39	IPOX positive for CD20, CD30, negative for CD3, CD15, EMA
YY-00-0302	Lymphoma	F	20	Hodgkins Lymphoma, IPOX positive CD30, CD15, CD20, status post treatment
YY-00-0297	Lymphoma	M	69	spleen, flow negative for CD5 and CD10

The tumor array (TARP3) was obtained from NIH TARP. The expression of IRF-7C was determined by the use of immunostaining technique. The additional information was obtained from the provider.

### IRF-7C Represses the Transactivation of IFN Promoter of IRF-7

**Figure 7:**
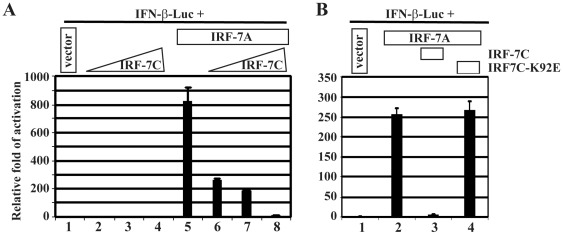
IRF-7C inhibits IRF-7-mediated transactivation of IFN promoter. A. IRF-7C inhibits the IRF-7-mediated activation of IFN-β reporter construct. 293T cells were transfected with 0, 50, 100, and 200 ng of IRF-7C (lanes 1–4 respectively) plus IFN-β promoter reporter construct and β-gal expression plasmids. Lanes 5–8, based on the same conditions of lanes 1–4, IRF-7A plasmid (100 ng) was added and also co-transfected into 293T cells with other plasmids. (B) DNA-binding of IRF-7C is required to inhibit the activation of IFN-β promoter. 293T cells were transfected with pcDNA3, IRF-7A (100 ng) IRF-7A+IRF-7C (200 ng), and IRF-7A+IRF7C-K92E (200 ng) respectively (lanes 1–4), along with IFN-β promoter reporter construct and β-Gal expression plasmids. Luciferase and β-gal activities were measured at 24 h after transfection. The relative folds of activation of promoter constructs are shown with standard deviations. One representative of three independent experiments is shown.

We next examined if IRF-7C is able to inhibit the transactivation activity of IRF-7 (IRF-7A). IRF-7 and IRF-7C were co transfected into 293T cells along with the IFN-β promoter reporter construct. IRF-7C was able to repress the transactivation function of IFN-β-promoter activity by IRF-7(Figure 7A). Also, the repression was associated with the ability of the IRF-7C to bind to DNA as the DNA-binding mutant of IRF-7C is unable to repress the activation (Figure 7B). In addition, IRF-7C was able to repress the Sendai virus induced activation of IFN-β promoter reporter construct (data not shown, also [41]). Thus, IRF-7C is able to block the transactivation function of IRF-7.

### IRF-7C Is Associated with Other IRF-7 Splicing Variants

**Figure 8:**
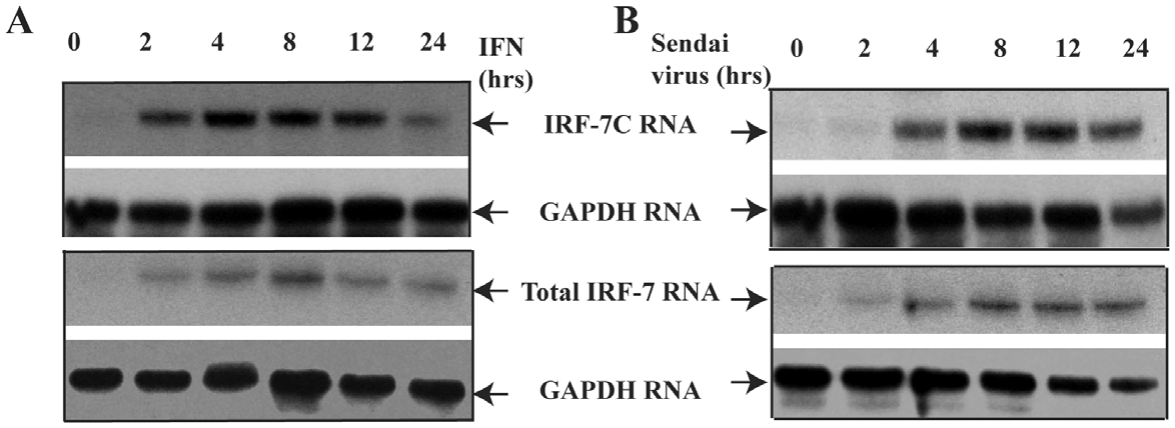
Correlative expression of IRF-7C and IRF-7. A. IRF-7 RNA expression upon IFN treatment. THP-1 cells were treated with IFN-α at 100 u/ml at the indicated times. Cells were collected at 0, 2, 4, 8, 12 and 24 hrs after treatment. Total RNAs were extracted with TRIzol (Invitrogen), and used for RPA with IRF-7C, IRF-7 (all splicing variants) and GAPDH probes. B. IRF-7 RNA expression upon virus infection. THP-1 cells were infected with Sendai virus, and cells were collected at 0, 2, 4, 8, 12 and 24 hrs post infection. Total RNAs were extracted with TRIzol (Invitrogen), and used for RPA. The images in the same box indicate that they are derived from the same gels. The identities of RNAs are shown.

We next examined the relation among the expression of IRF-7C and other IRF-7 splicing variants. With limited experiments, we did not observe any splicing-specific regulation of IRF-7C by EBV LMP-1 (data not shown). However, it is well-known that type I IFN as well as viral infection induces the expression of IRF-7. We thus examined the expression of IRF-7C and its relation to other IRF-7 splicing variants during those induction processes. THP-1 cells were treated with IFN-α or Sendai virus. Cells were collected and RNA was isolated at various times. The same RNAs were used for RPA for the detection of IRF-7C as well as IRF-7(all variants) respectively. As shown in Figure 8, IRF-7C is associated with other IRF-7 splicing variants, and no obvious splicing-specific expression was observed in either IFN-α or Sendai virus treated cells.

## Discussion

In this report, we have examined the possible role of the IRF-7C, one splicing variant of IRF-7, in EBV transformation of human B lymphocytes. First of all, because IRF-7C mRNA structure apparently fits the two hallmarks for NMD degradation [39], it is thus necessary to test the existence of IRF-7C under physiological conditions. Our data clearly indicate that IRF-7C protein is indeed expressed in cells both in pathogenic and physiological conditions. IRF-7C RNA is expressed proportionally to other IRF-7 variants' RNA in several induction conditions. Thus, NMD-mediated mRNA decay seems not a major player in the regulation of IRF-7C expression.

With the IRF-7C specific antibody, we have found that IRF-7C is associated with EBV transformation in primary B cells (Figure 3). The basis for the association is apparently related to the fact that IRF-7C is associated with EBV Type III latency (Figures 1 and 2), and EBV LMP-1 is at least partially responsible for the induction of IRF-7C in B cells (Figure 4). Because LMP-1 is required for EBV transformation, and LMP-1 is a membrane protein that requires cellular proteins for its signaling and transformation functions, it is thus suggested that IRF-7C is involved in the EBV-mediated transformation of primary B cells. Furthermore, IRF-7C is able to transform NIH 3T3 cells (Figure 5A), and partially rescue IRF-4-knockdown-mediated cell growth inhibition (Figure 5B). Because specific knockdown of endogenous IRF-7 in EBV-transformed cells has not been established yet, the direct role of IRF-7C in EBV transformation without other IRF-7 splicing variants is still not available. However, all these data collectively suggest that IRF-7C has oncogenic potential and might be a factor in EBV transformation process.

We also examined the expression of IRF-7C in a tumor array that contains more than 400 different primary tumor specimens, and our data indicated that several lymphoma tissues expressed IRF-7C extensively (Figure 6 and Table 1). Because LMP-1 expression is sporadic in tumor cells, the connection between LMP-1 and IRF-7C in vivo is not clearly established (data not shown). Sporadic IRF-7C-espression cells in some other tumors are also observed. Thus, IRF-7C might also play a role in the development of other tumors, not necessarily only associated with EBV.

IRF-7C has the DNA binding domain only [23]. It is known that the DNA binding domain of IRF-2 alone is able to transform at least rodent cells, possibly through competition with other IRFs for binding to the same or similar DNA sequences [42]. It is thus possible that IRF-7C is also using the similar mechanism. This notion is supported by the fact that the IRF-7C-mutant, IRF7C-K92E, failed to rescue the IRF4-knockdown-mediated cell growth inhibition (Figure 5B).

IRF-7C is associated with other IRF-7 variants in EBV transformed cells (Figures 1 and 2; Also data not shown, and references [26], [30], [37]), as well as in IFN-treated or Sendai virus-infected cells (Figure 8). Thus, IRF-7C is apparently associated with IRF-7 in those situations examined without a specific splicing-specific expression. This close association among IRF-7C and other IRF-7 variants suggests that IRF-7C and IRF-7 may collectively contribute to the transformation processes of EBV. Other than in viral transformation, IRF-7C may contribute to the regulation of both viral and cellular promoters, notably EBV Qp. IRF-7C inhibits the promoter activity of Qp [23]. The expression of IRF-7C is inversely associated with Qp activity (Qp is inactive in type III latency, but Qp is active in type I latency) [23]. Thus, both IRF-7 and IRF-7C may contribute the inactivation of Qp in type III latency.

IRF-7C is able to inhibit the function of IRF-7 in transactivation IFN promoter reporter constructs (Figure 7). Given the facts that: 1) IRF-7 is highly expressed and activated in EBV transformed cells [21], [23], [43]; 2) activated (or over-expression of) IRF-7 may activate endogenous type I IFNs [41], [44], [45]; and 3) EBV-transformed cells do not produce type I IFNs [13], [14], it is possible that IRF-7C could be used by the cells to block the spontaneous IFN production during EBV transformation. This might be important for viral transformation as IFNs are a factor in controlling the transformation process: EBV transformation is inhibited by IFNs and the virus encodes genes that specifically nullify the functions of IFNs [46], [47], [48], [49], [50].

Furthermore, it is well known that IFN treatment increases IRF-7 levels [45], [51], [52], and IFN-treated cells do not produce type I IFNs [53], [54], [55]. Therefore, IRF-7C induced by IFN-treatment might play a similar role in repressing spontaneous IFN production processes during IFN treatment. Unfortunately, due to the fact that a splicing-specific knockdown of IRF-7C is not available, this notion is hard to verify experimentally.

Thus, our data collectively suggest that EBV may use splicing variants of IRF-7 for its transformation process in two strategies: to use the oncogenic properties of various IRF-7 splicing variants to promote transformation process, but uses one of its splicing variants (IRF-7C) to block the detrimental function of IRF-7 in IFN-induction. In addition to IRF-7C, other IRFs, such as IRF-4, might also be involved in the inhibition of IFN processes. IRF-4 is a known inhibitor for IFN production, and a known key player in EBV transformation [40].

IRFs and EBV transformation have close relations. IRFs are used by EBV to regulate both viral and cellular factors that are involved in EBV latency and transformation [14], [26], [37], [43], [56], [57]. Other than IRF-7, IRF-4 and IRF-5 are also associated with EBV transformation. IRF-5, likely a tumor suppressor [58], [59], [60], [61] [62], is highly expressed in EBV transformed cells and, together with IRF-4, may be involved in the EBV-mediated regulation of Toll-like receptor 7 activities [56]. Thus it is apparent that EBV induces a balanced expression of IRFs during EBV transformation. With reciprocal inhibition and/or activation among these factors, EBV may lead the infected cell to apoptosis or proliferation for its own benefits in various microenvironments for the survival of the virus in vivo. The work here has expanded our knowledge about IRFs and EBV transformation and provides more details in the potential balanced actions among various IRFs in EBV transformation processes.
